# A prospective, single centre, open label, single arm pilot study to evaluate the efficacy and safety of Amlapitta Mishran Suspension in participants with endoscopic gastritis

**DOI:** 10.1016/j.jaim.2022.100664

**Published:** 2022-11-24

**Authors:** Yashashri C. Shetty, Paresh G. Koli, Manoj Lahoti, Savita Kulkarni, Preeti Rajput, Mukesh B. Chawda

**Affiliations:** a Department of Pharmacology and Therapeutics, Seth GS Medical College and KEM Hospital, Mumbai, Maharashtra, India; b Department of Hepatology & Gastroenterology, Suyash Hospital, India; c Consulting Ayurved Physician Suyash Hospital, India; d Medical Services, Shree Dhootapapeshwar Limited, India

**Keywords:** Amlapitta, Gastritis, Gastroenterology, Ayurved

## Abstract

**Background:**

Endoscopic gastritis is associated with symptoms of gastritis, along with endoscopic findings. Amlapitta Mishran has multiple active components that act via various mechanisms in patients with gastritis symptoms. We planned to conduct this study to find out the efficacy and safety of Amlapitta Mishran in patients with endoscopic gastritis.

**Objectives:**

To find out efficacy of Amlapitta Mishran in patient with endoscopic gastritis.

**Materials and methods:**

This study was an open-label, prospective, single-center study. Thirty participants were recruited, and Amlapitta Mishran Suspension was given for 30 days. Blood investigations for safety were performed at baseline (Visit 1), on Visit 3 and Visit 4. Endoscopy was performed at baseline and Visit 4, and stomach erosion score was recorded. Amlapitta Symptom Rating Scale score, Postprandial Distress Syndrome (PPDS) score, and Epigastric Pain Syndrome (EPS) score were efficacy endpoints.

**Results:**

Out of the 30 participants recruited, 28 participants completed the study. The median age of participants in the study was 26.50 years. A statistically significant (P<0.05) reduction was seen in endoscopy score at Visit 4 as compared to baseline (Visit 1) by Wilcoxon Signed Rank test. Amlapitta Symptom Rating Scale score, PPDS score, EPS score also exhibited significant reduction (P < 0.05) at Visit 3 and Visit 4 as compared to baseline by Friedman’s test with post hoc analysis. No statistically significant reduction was seen in these scores from Visit 3 to Visit 4, except for the EPS score. At the end of Visit 4, 18 (64%) participants had an endoscopy score of 1 (no erosions). At the end of Visit 4, ≥ 50% improvement was seen in Amlapitta Symptom Rating Scale score in 27 (96%) participants, PPDS score improved by ≥ 50% in 25 (89%) participants, and EPS score improved by ≥ 50% in 26 (93%) participants. All safety variables including laboratory investigation were within the normal range in all visits.

**Conclusion:**

Amlapitta Mishran Suspension effectively reduced endoscopic gastritis scores in the participants and reduced the symptoms of gastritis measured by the Amlapitta Symptom Rating Scale, PPDS, and EPS scores with no adverse events.

## 1 Introduction

Gastritis is a common endoscopic diagnosis and can range from asymptomatic to severely symptomatic gastritis and can be acute or chronic. When the patient develops dyspeptic symptoms and there is no other explanation found for the symptoms, patient undergoes an upper gastrointestinal endoscopy and the endoscopic diagnosis of gastritis is usually made [1].

Gastritis symptoms are non-specific and therefore other causes of symptoms are excluded before diagnosing gastritis. The gastric mucosa is protected by a layer of thick mucosa which acts as the first line of defense against gastric acid in the lumen. Alcohol and some drugs disrupt this layer, exposing gastric mucosa to acid or alkali attack, which results in gastritis. The common symptoms include epigastric discomfort, nausea, and early satiety. In case of obvious etiology, the removal of cause is the priority. Conventional management is directed towards a reduction in acid-secretion of gastric mucosa, mucosal barrier protection, and gastric prokinetic agents. Anti-secretory agents like H_2_ receptor antagonists or Proton Pump Inhibitors [PPIs] or cytoprotective drugs (misoprostol, sucralfate, Aluminium ions, etc.) can facilitate the repair of the gastric mucosal barrier [[1], [2], [3]].

Endoscopy helps in differentiating gastritis from Functional Dyspepsia, which is not associated with endoscopic abnormality [2]. Functional Dyspepsia includes all the symptom complex of gastritis. Functional Dyspepsia (FD) is divided into two sub-groups according to cardinal symptoms viz.: Postprandial Distress Syndrome (PPDS) and Epigastric Pain Syndrome (EPS) [3].

Acid suppressive drugs are mainly used for the EPS subgroup. Prokinetic agents are used to restoring motility abnormalities associated with PDS. In case of insufficient symptom control, acid-suppressive drugs can be complemented with a prokinetic or vice versa. Neuromodulators (antidepressants and anxiolytics) in low doses are also used. Phytotherapy (Peppermint oil and Caraway oil) or nonpharmacological treatment options (psychotherapy or acupuncture) can be added as complementary therapy [3].

Although modern medicine offers many medicaments for the management of Acid Peptic Disorders like gastritis, their efficacy and safety may be a cause of concern [4]. Thus, there is a need for a new drug with a lower side effect profile and good efficacy. Ayurved the ancient Indian system of medicines is a rich database of herbal, mineral, and animal origin raw materials/ingredients for the management of acid peptic disorders. Amlapitta is the nearest correlate to acid peptic disorders, mainly gastritis, and many Ayurved ingredients are extensively studied and documented for their acid-neutralizing, cytoprotective, and ulcer healing potential.

Amlapitta Mishran Suspension is widely used by Ayurved and allopathic Practitioners across India in the management of Acid-Peptic Disorders. Amlapitta Mishran Suspension is documented for its anti-ulcer effect in an experimental model of indomethacin-induced gastric ulcers in rats [5]. It has multiple ingredients with different actions that act in synergy and these ingredients possess anti-emetic, anti-ulcer, anti-secretory, gastroprotective, and acid-neutralizing properties [[6], [7], [8], [9], [10], [11], [12], [13], [14], [15], [16], [17], [18]].

There are several proprietary formulations for the management of Acid Peptic Disorders, but Amlapitta Mishran Suspension is unique in its components and has a strong pharmacopoeial base. It offers benefits because of multiple ingredients. The important attributes of ingredients of Amlapitta Mishran Suspension are:

• Vasa (*Adhatoda vasica*) possesses anti-emetic [[7], [19]] and anti-ulcer effect [7].
• Guduchi (*Tinospora cordifolia*) has a documented antiulcer activity in the experimental model, as evidenced by a reduction in ulcer index along with the decrease in gastric volume, total acidity, and an increase in pH of gastric content [8].
• Parpata (*Fumaria indica*) possess antisecretory (inhibition of acid secretion), gastroprotective (potentiation of defensive factors), and in-vitro antacid activity [9].
• Nimba (*Azadirachta indica*) bark extract exhibited therapeutic potential for controlling gastric hypersecretion and gastroesophageal and gastroduodenal ulcers in humans [10].
• Kiratatikta (*Swertia chirata*) is reported to be effective in experimentally induced gastric ulcers in rats [11].
• Bhrungaraja (*Eclipta alba*) extract possesses potent antisecretory and gastroprotective activity [12].
• Patola (*Trichosanthes dioica*) extract significantly increased the pH of gastric acid, reduced the volume of gastric juice and pepsin activity showing its antiulcer potential [13].
• Yashti (*Glycyrrhiza glabra*) aqueous extract is reported for its anti-ulcerogenic potential in an experimental model of stress-induced gastric ulcer in Wistar rats [14]. It is also reported for its healing effect in *Helicobacter pylori* infected peptic ulcers [15].
• Haritaki (*Terminalia chebula*), Bibhitaka (*Terminalia belerica*), Amalaki (*Emblica officinalis*) in combination are termed as *Triphala*, these herbs individually and in combination are documented to possess anti-ulcer and cytoprotective effect [16,17].
• Shouktik (Muktashukti) Bhasma [Processed Seashell] has an acid-neutralizing effect and showed significant anti-ulcer activity in the aspirin-induced ulcer model in rats [18].

Each 10 ml of Amlapitta Mishran contains Vasa (A. vasica) leaf 100 mg, Guduchi (*T. cordifolia*) stem 100 mg, Parpata (*F. indica*) Whole Plant 100 mg, Nimba (*A. indica*) Stem Bark 100 mg, Kiratatikta (*S. chirata*) Whole Plant 100 mg, Bhrungaraja (*E. alba*) Whole Plant 100 mg, Patola (*T. dioica*) Leaf 100 mg, Yashti (*G. glabra*) Stem & Root 100 mg, Haritaki (*T. chebula*) Fruit Pericarp 33.33 mg, Bibhitaka (T. belerica) Fruit Pericarp 33.33 mg, Amalaki (E. officinalis) Fruit Pericarp 33.33 mg, and Shouktik Bhasma (Processed Seashell) 500 mg. Along with the ingredients, there are multiple phytochemical components of the ingredients which could be contributing to the effectiveness of Amlapitta Mishran Suspension [16]. Amlapitta Mishran Suspension composition is ingredients of Bhunimbadi Kwath + Yashti (*G. glabra*) + Shouktik Bhasma. Since the base formulation Bhunimbadi Kwath is in the form of liquid and addition of Shouktik Bhasma, which is insoluble in water, would settle down in the bottle; this formulation was developed in the form of a suspension. Also, the dosage form in the form of liquid may provide faster symptomatic relief.

Also, there are no clinical trials done on Amlapitta Mishran. With this background, we planned this study to find out the effectiveness and safety of Amlapitta Mishran Suspension in participants with endoscopic gastritis.

## 2 Methods

This study was an open-label, prospective, single-center study. One-week period before the treatment allocation was for participant arrival, screening, enrolment in the study, informed consent, study procedure, and observation. After Ethics Committee approval and subsequent CTRI registration (CTRI/2020/02/023,224), this study was started, recruitment was done over 4 months. The entire duration of the study was 9 months (Feb 2020 to November 2020), including analysis. The duration of the study participation for each participant was 30 days for test medication. The study was conducted at OPD of Medical Gastroenterology.

Participants recruited in the study were of either gender in the age group of 18–65 years, with endoscopic gastritis diagnosed by the endoscopic erosion scores, negative Rapid Urease Test (RUT), normal ECG, willing to consent, and follow-up. Participants who were pregnant or lactating, previously received Amlapitta Mishran Suspension, with documented *H. pylori* infection, contraindications to undergo endoscopy, medical/surgical conditions (Zollinger Ellison Syndrome, Gastrectomy etc.) which can interfere with study results, abnormal blood investigation, had taken study drug or any oral herbal medication in the past 4 weeks, and participants administered with H2-receptor antagonists, muscarinic receptor antagonists, gastrin receptor antagonists, proton pump inhibitors, prostaglandin analogs or mucosal protective agents before study in 1 week time were excluded from the study. Dietary and lifestyle changes (avoiding spicy and oily food, alcohol, smoking etc.) were recommended to all the participants.

Visit 1 and 2 (Day 1) were planned on the same day for the convenience of the participants. Visit 3 was on Day 15 ± 3 days and Visit 4 was on Day 30 ± 3 days.

Participants attending the OPD with a history of gastritis, satisfying the selection criteria were selected. Baseline Amlapitta Symptoms Rating Scale score, Post Prandial Distress Syndrome (PPDS) score, and Epigastric Pain Syndrome (EPS) score were assessed. Stomach erosions were assessed with the help of the Gastrointestinal endoscopy at baseline. Blood investigations like CBC (Complete Blood Count), LFT (Liver Function Tests), RFT (Renal Function tests), HbA1c) were done at baseline and visit 4 to assess safety. Recruitment, assessment, and follow-up was done by the investigators working at the hospital.

Study Medication was Amlapitta Mishran Suspension (marketed by Shree Dhootapapeshwar Limited) taken in a dose of 15 ml before meals twice daily for 30 days. On the baseline visit (Visit 1 & 2) and follow-up visit (Visit 3), the participants were given Amlapitta Mishran Suspension (3 bottles of 200 ml each).

Participants who withdrew consent had <80% compliance for study medication. Patients who worsened/no improvement of symptoms after 7 days of treatment was seen were discontinued from the study.

Gastroenterologist performing and scoring the endoscopy was blinded about the treatment to the participant. Stomach Erosions Scores were measured with the help of the Gastrointestinal endoscopy. Scores were No Erosion (Normal, score 1), 1–2 erosions (Mild, score 2), 3–5 erosions (moderate, score 3), and 6 or more erosions (severe, score 4).

Amlapitta Symptom Ratings Scale (Total score Range 0–21) had components (each has sub-score of 3) like Avipaak (Indigestion), Klama (Tiredness), Utklesha (Nausea), Tikta amla Udgaar (Sour and bitter belching), Guruta (Feeling of heaviness in the body), Hrit-Kantha Daha (Burning sensation), and Aruchi (Anorexia).

Postprandial Distress Syndrome (PPDS) score (Bothersome postprandial fullness severe enough to affect usual activities and Bothersome early satiation severe enough to prevent finishing a regular-size meal) and Epigastric Pain Syndrome (EPS) score (Bothersome epigastric pain severe enough to affect usual activities and Bothersome epigastric burning, severe enough to affect usual activities) were measured on Likert scale (scores 1–5).

Outcome measures were to show improvement of endoscopic gastritis score, Amlapitta symptom rating score, PPDS score, and EPS score.

In this study, a sample size of 30 study participants was taken. As it was a pilot and first study in humans, therefore no formal sample size calculation. Data were analyzed using Microsoft Excel and SPSS trial version 25 software. Descriptive statistics were used. Data expressed in percentages and mean ± SD and Median. The normality of data was assessed using the Shapiro–Wilk test. Within-group associations for over 2-time points were checked with Friedman's test, followed by a post hoc test for intergroup comparisons. Within-group comparisons for 2-time points were analyzed by Wilcoxon Signed rank test. The level of significance in the study was 0.05.

## 3 Results

**Fig. 1:**
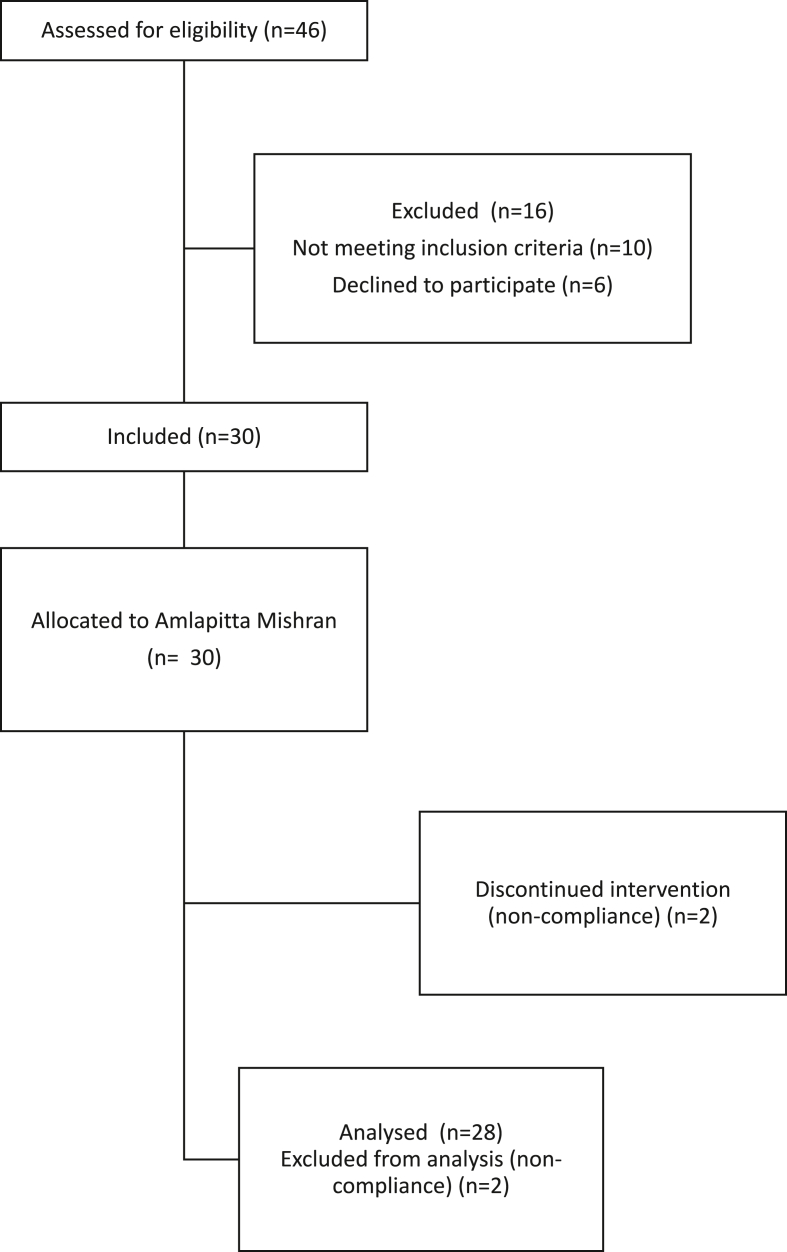
Patient enrollment flowchart.

Out of the 30 participants recruited for the study, 28 participants completed the study. Two participants were considered as drop-out at Visit 4 by the Physician for non-compliance to study medications, therefore the final data comprises the data of 28 participants. For logistics reasons, the workup of Visit 1 and Visit 2 was performed on the same day. For the analysis, visit 1 is counted as the baseline (Fig. 1).

Out of 30 participants, 20 were female and 10 were male. The mean age was 27.43 ± 7.50. Mean HbA1c was 5.08 ± 0.35. There was no statistically significant difference in laboratory values from baselines (Visit 1) except systolic blood pressure, serum indirect bilirubin, Blood Urea Nitrogen (BUN), and serum creatinine (see Table 1).

**Table 1:** Comparison of laboratory values.

Laboratory Values	Visit 1	Visit 3	Visit 4	P (V1 vs V3)	P (V1 vs V4)	P (V3 vs V4)
Temperature	35.98 ± 0.24	35.99 ± 0.34	35.94 ± 0.29	>0.05	>0.05	>0.05
Pulse Rate/min	81.93 ± 5.95	83.57 ± 8.23	83.79 ± 6.78	>0.05	>0.05	>0.05
Respiratory Rate/min	19.86 ± 1.21	20.36 ± 1.81	20.21 ± 1.75	>0.05	>0.05	>0.05
Systolic Blood Pressure	111.43 ± 10.44	105.71 ± 9.2	107.14 ± 8.1	<0.05∗	>0.05	>0.05
Diastolic Blood Pressure	71.43 ± 8.03	68.93 ± 6.85	69.29 ± 6.04	>0.05	>0.05	>0.05
Hemoglobin	12.78 ± 0.89	12.95 ± 0.87	13.43 ± 0.91	>0.05	>0.05	>0.05
RBC's	4.7 ± 0.5	4.57 ± 0.48	4.52 ± 0.52	>0.05	>0.05	>0.05
WBC's	8144.82 ± 2213.67	7691.86 ± 2212.85	7179.44 ± 2232.84	>0.05	>0.05	>0.05
Polymorphs	64.21 ± 5.75	61.64 ± 7.68	60.39 ± 7.86	>0.05	>0.05	>0.05
Lymphocytes	26.93 ± 6	29.64 ± 7.02	29.57 ± 8.12	>0.05	>0.05	>0.05
Monocytes	6.93 ± 2.21	7.14 ± 2.09	7.64 ± 1.59	>0.05	>0.05	>0.05
Eosinophils	1.76 ± 0.54	1.76 ± 0.54	2.19 ± 0.98	>0.05	>0.05	>0.05
Basophils	0 ± 0	0.04 ± 0.19	0 ± 0	>0.05	>0.05	>0.05
Platelets	2.73 ± 0.72	2.68 ± 0.85	2.58 ± 0.94	>0.05	>0.05	>0.05
AST	17.82 ± 5.66	17.64 ± 6.72	15.18 ± 2.84	>0.05	>0.05	>0.05
ALT	22.39 ± 5.53	23.18 ± 6.71	20.18 ± 4.98	>0.05	>0.05	>0.05
Serum Bilirubin Direct	0.15 ± 0.06	0.2 ± 0.14	0.14 ± 0.07	>0.05	>0.05	>0.05
Serum Bilirubin Indirect	0.19 ± 0.09	0.28 ± 0.11	0.21 ± 0.1	<0.05∗	<0.05∗	>0.05
BUN	8.58 ± 2.68	12.68 ± 4.2	12.11 ± 3.75	<0.001∗	>0.05	>0.05
Serum Creatinine	1.11 ± 1.75	1.5 ± 2.65	0.85 ± 0.13	<0.05∗	>0.05	>0.05

V1 is Visit 1, V3 is Visit 3, and V4 is Visit 4.

∗Statistically significant (P < 0.05) on Friedman's test with the post hoc test for within-group comparisons.

The mean endoscopy score at Visit 1 was 3.57 ± 0.50 which significantly reduced to 1.36 ± 0.49 (P < 0.05) at Visit 4. At Visit 1, 16 (57%) and 12 (43%) participants had endoscopy score 4 (6 or more erosions - Severe) and score 3 (3–5 erosions - Moderate) respectively. At Visit 4, 18 (64%) participants had endoscopy score 1 (no erosions - Normal) and 10 (36%) participants had endoscopy score 2 (1–2 erosions - Mild) (see Table 2).

**Table 2:** Endoscopy and Symptoms scales comparison.

	Mean	SD	25th	50th	75th	P (v/s Visit 1)	P (v/s Visit 3)
Amlapitta Scale Score Visit 1	9.25	3.11	6.25	9.00	12.75		
Amlapitta Scale Score Visit 3	4.07	1.72	3.00	4.00	5.75	<0.001^#^	
Amlapitta Scale Score Visit 4	2.50	1.17	2.00	2.00	3.00	<0.001^#^	>0.05
Endoscopy Score Visit 1	3.57	0.50	3.00	4.00	4.00		
Endoscopy Score Visit 4	1.36	0.49	1.00	1.00	2.00	<0.001∗	
Bothersome Post-Prandial Fullness Visit 1	2.82	0.72	2.00	3.00	3.00		
Bothersome Post-Prandial Fullness Visit 3	1.54	0.58	1.00	2.00	2.00	<0.001^#^	
Bothersome Post-Prandial Fullness Visit 4	1.11	0.50	1.00	1.00	1.00	<0.001^#^	>0.05
Bothersome Early Satiation Visit 1	2.46	0.69	2.00	2.00	3.00		
Bothersome Early Satiation Visit 3	1.25	0.52	1.00	1.00	1.00	<0.001^#^	
Bothersome Early Satiation Visit 4	1.04	0.58	1.00	1.00	1.00	<0.001^#^	>0.05
PPDS Score Visit 1	5.29	1.30	4.00	5.00	6.00		
PPDS Score Visit 3	2.79	0.88	2.00	3.00	3.00	<0.001^#^	
PPDS Score Visit 4	2.14	0.71	2.00	2.00	3.00	<0.001^#^	>0.05
Bothersome Epigastric Pain Visit 1	3.21	0.74	3.00	3.00	4.00		
Bothersome Epigastric Pain Visit 3	1.86	0.52	2.00	2.00	2.00	<0.001^#^	
Bothersome Epigastric Pain Visit 4	1.32	0.48	1.00	1.00	2.00	<0.001^#^	>0.05
Bothersome Epigastric Burning Visit 1	3.39	0.74	3.00	4.00	4.00		
Bothersome Epigastric Burning Visit 3	1.71	0.60	1.00	2.00	2.00	<0.001^#^	
Bothersome Epigastric Burning Visit 4	1.21	0.42	1.00	1.00	1.00	<0.001^#^	>0.05
EPS Score Visit 1	6.61	1.31	6.00	6.00	8.00		
EPS Score Visit 3	3.57	0.92	3.00	4.00	4.00	<0.001^#^	
EPS Score Visit 4	2.54	0.58	2.00	2.50	3.00	<0.001^#^	<0.05^#^

50th percentile is median. 25th and 75th percentile are interquartile range.

∗Statistically significant (P < 0.05) on Wilcoxon Signed Rank test for Endoscopy Score, and.

# Statistically significant (P < 0.05) on Friedman's test with the post hoc test for within-group comparisons.

The mean Amlapitta Symptom Rating Scale score at Visit 1 (Baseline) was 9.25 ± 3.11 which significantly (P < 0.05) reduced to 4.07 ± 1.72 and 2.50 ± 1.17 at Visit 3 and Visit 4, respectively. The reduction in the score at Visit 4 was not statistically significant as compared to the Visit 3 score. 24 (85.71%) participants and 27 (96.43%) participants exhibited more than or equal to 50% reduction in Amlapitta Symptom Rating Scale score at Visit 3 and Visit 4 respectively as compared to Visit 1 (Baseline).

The mean Postprandial Distress Syndrome score at Visit 1 (Baseline) was 5.29 ± 1.30 which significantly (P < 0.05) reduced to 2.79 ± 0.88 and 2.14 ± 0.71 at Visit 3 and Visit 4, respectively. The reduction in the score at Visit 4 was not statistically significant as compared to the Visit 3 score. 15 (53.57%) participants and 25 (89.29%) participants exhibited more than or equal to 50% reduction in Postprandial Distress Syndrome score at Visit 3 and Visit 4 respectively as compared to Visit 1 (Baseline).

Both the variables of Postprandial Distress Syndrome Viz. Bothersome Postprandial Fullness and Bothersome Early Satiation exhibited statistically significant (P < 0.05) reduction in respective scores at Visit 3 and Visit 4 as compared to the baseline (Visit 1) score.

The mean Epigastric Pain Syndrome score at Visit 1 (Baseline) was 6.61 ± 1.31 which significantly (P < 0.05) reduced to 3.57 ± 0.92 and 2.54 ± 0.58 at Visit 3 and Visit 4, respectively. The reduction in the score at Visit 4 was also statistically significant (P < 0.05) as compared to the score at Visit 3.

16 (57.14%) participants and 26 (92.86%) participants exhibited more than or equal to 50% reduction in Epigastric Pain Syndrome score at Visit 3 and Visit 4 respectively as compared to Visit 1 (Baseline).

Both the variables in Epigastric Pain Syndrome Viz. Bothersome Epigastric Pain and Bothersome Epigastric Burning exhibited a statistically significant (P < 0.05) reduction in respective scores at Visit 3 and Visit 4 as compared to Baseline (Visit 1) score.

## 4 Discussion

In our study, we found that Amlapitta Mishran suspension improved the endoscopy scores, and reduced symptoms measured by Amlapitta Symptom Rating Scale score, PPDS Score, and EPS Score.

An endoscopic diagnosis of gastritis is very common. The patients having significant symptoms associated with endoscopic findings and no other explanation/cause for their symptoms are usually/commonly recommended Proton Pump Inhibitors [PPIs] [1].

The pharmacological treatment of patients with gastritis using acid suppressants usually achieves only partial symptomatic relief in most of the cases and of late the role of the cytoprotective agents in strengthening the mucosal defensive factors is gaining importance [21]. It is assumed that these drugs ultimately balance the aggressive factors (acid, pepsin, *H. pylori,* and bile salts) and defensive factors (mucin secretion, cellular mucus, bicarbonate secretion, mucosal blood flow, and cell turnover) [21]. The logical treatment strategy in patients with symptomatic gastritis is the combination of acid suppression and mucosal protection [1].

In our study, the endoscopy score was reduced from 3.57 ± 0.50 at baseline to 1.36 ± 0.49 at Visit 4, which is a significant reduction in the endoscopy score showing the effectiveness of Amlapitta Mishran Suspension in reducing endoscopic gastritis. After 30 days of treatment 18 (64%) participants had endoscopy score 1 (no erosions - Normal) and 10 (36%) participants had endoscopy score 2 (1–2 erosions - Mild).

Gastritis occurs because of an imbalance in offensive factors (gastric acid) and defensive factors (mucosal protection). Amlapitta Mishran Suspension acts by reducing the offensive factors like reducing acid secretion, neutralizing the acid, and reduced pepsin activity. Also, it acts on defensive factors by accelerating the healing of erosions/ulcers because of its cytoprotective effect. These effects are because of the synergistic action of multiple components of Amlapitta Mishran Suspension.

In a 2010 study by Dewan et al. [21], Troxipide was compared with Ranitidine for endoscopic gastritis. Troxipide lead to a reduction in gastric mucosal erosion, oozing, redness, and edema scores, and Ranitidine also reduced these scores. The reduction in gastric mucosal erosion with Troxipide and Ranitidine was 98.31% and 78.18% respectively after 4 weeks of therapy. The percentage of participants showing complete symptom resolution (abdominal pain, bloating, belching, nausea, and heartburn) in participants with endoscopic gastritis was more in the Troxipide group as compared to the Ranitidine group.

In our study, we also observed a reduction in the symptom scores on parameters like the Amlapitta Symptoms Rating Scale score, PPDS score, and EPS score. Out of 28 participants, who completed the study, 27 (96.43%) participants exhibited more than or equal to 50% reduction in Amlapitta Symptom Rating Scale score after 30 days of treatment. 25 (89.29%) participants and 26 (92.86%) participants exhibited more than or equal to 50% reduction in Postprandial Distress Syndrome score Epigastric Pain Syndrome score respectively after 30 days of treatment with Amlapitta Mishran Suspension. Gastritis can resolve spontaneously if the offending agent is removed or based on the improved diet and exercise in the patients. This could be the reason for the improvement in our study apart from the medication.

Gastritis is a commonly treated condition by general practitioners, and in most cases, they do not rely on endoscopy for diagnosis and treatment of gastritis. They treat gastritis based on signs and symptoms. The reduction in Amlapitta Symptoms Rating Scale score, PPDS score, and EPS score exhibited by Amlapitta Mishran Suspension reconfirms its potential to offer a symptomatic improvement in acute and chronic gastritis. It can also be used for the relief in symptom complex associated with Functional Dyspepsia.

We used the Amlapitta Symptom Rating Scale as it is commonly used in the studies on Amlapitta [22,23]. EPS and PPDS scores are widely used scores for functional dyspepsia and are validated [24,25]. They include all the symptom complex of gastritis and therefore these scores were used to assess symptomatic improvement. Endoscopic gastritis includes endoscopic abnormalities in the gastric mucosa and symptoms which are measured by EPS and PPDS which are part of functional dyspepsia symptoms.

In a 2021 study, Urdhwaga Amlapitta was treated with 96% cure rate with Kamdudha ras, Giloy satva, Shankh bhasm, Avipattikar churn, and Chitrakadi vati [3].

This study was of 30 days duration because any drug which works against gastritis heals the lesions in 30 days, so the duration was selected. Amlapitta Mishran Suspension exhibited clinical efficacy at Visit 3 (Day 15) which shows its early onset of the action with multiple ingredients working in unison with different mechanisms.

The only study done on Amlapitta Mishran Suspension is an animal study to evaluate the anti-ulcer effect. Amlapitta Mishran Suspension treated rats showed a significant (P < 0.0001) decrease in the total number of ulcers and ulcer index and a significant increase in % inhibition of ulcers as compared with Ranitidine, which was a positive control group [5].

This was the first human study on Amlapitta Mishran Suspension. It is marketed in India in the management of Amlapitta (Acid Peptic Disorders like gastritis) for over 18 years of age. It is the most prescribed and preferred Ayurved medication for the management of Amlapitta amongst the Ayurved fraternity in India. Till date, no adverse events have been reported/complaint has been received for this product.

Participants in our study were evaluated for the exclusion criteria by investigations (rapid urease test, endoscopy, blood investigations like CBC, LFT, RFT), and history of other diseases/medication uptake was found out by looking at the patient's hospital history files and by directly asking the appropriate questions to the patient. As this was a first in human study of this kind, exclusion criteria were kept strict to minimize the bias.

Also, Amlapitta Mishran Suspension was safe in all the participants as there were no adverse events (AEs) and serious adverse events (SAEs) reported. There were no safety issues with laboratory investigations as well. The statistically significant changes in the laboratory variables were not clinically significant, as they were in the reference range.

The limitations of our study were that it was a single-arm study, we could not compare it with the available treatment options. Because of the open-label design, there could be a bias from both participants' and physicians’ side in the description of symptoms or evaluation. As it was a single-center study conducted by a single gastroenterologist, there could be a bias in recruitment, follow-up, and assessment. As the study molecule is ayurvedic but the partial methodology and the variables are according to allopathy. Ayurvedic pathophysiology and ayurvedic concepts were partially used for developing the methods. Investigators prescribed ayurvedic preparation without making ayurvedic diagnosis for research purpose. Gastritis can resolve on its on in some patients if the offending agent is removed.

## 5 Conclusion

**Amlapitta Mishran Suspension** effectively reduced endoscopic scores in the participants with Endoscopic gastritis and reduced the clinical symptoms of gastritis measured by the Amlapitta Symptom Rating Scale, Postprandial Distress Syndrome (PPDS) and Epigastric Pain Syndrome (EPS) scores, while being a safe medication with no adverse events.

## Source(s) of support

Shri Dhootapapeshwar Limited.

## Presentation at a meeting

Nil.

## CRediT author statement

Yashashri C. Shetty, Paresh G. Koli, Manoj Lahoti and Mukesh B. Chawda – Conceptualization, Methodology / Study design, Software, Validation, Formal analysis, Investigation, Resources, Data curation, Writing – original draft, Writing – review and editing, Visualization, Supervision, Project administration, Funding acquisition. Savita Kulkarni and Preeti Rajput - Methodology / Study design, Investigation, Resources, Visualization, Supervision, Project administration.

## Conflict of interest

Nil.
